# High shear resistance of insect cells: the basis for substantial improvements in cell culture process design

**DOI:** 10.1038/s41598-021-88813-4

**Published:** 2021-05-03

**Authors:** Florian Strobl, Mark Duerkop, Dieter Palmberger, Gerald Striedner

**Affiliations:** 1 ACIB GmbH, Vienna, Austria; 2 Institute of Bioprocess Science and Engineering, University of Natural Resources and Life Sciences, Vienna, Austria; 3 Novasign GmbH, Vienna, Austria

**Keywords:** Biotechnology, Cell biology, Physiology

## Abstract

Multicellular organisms cultivated in continuous stirred tank reactors (CSTRs) are more sensitive to environmental conditions in the suspension culture than microbial cells. The hypothesis, that stirring induced shear stress is the main problem, persists, although it has been shown that these cells are not so sensitive to shear. As these results are largely based on Chinese Hamster Ovary (CHO) cell experiments the question remains if similar behavior is valid for insect cells with a higher specific oxygen demand. The requirement of higher oxygen transfer rates is associated with higher shear forces in the process. Consequently, we focused on the shear resistance of insect cells, using CHO cells as reference system. We applied a microfluidic device that allowed defined variations in shear rates. Both cell lines displayed high resistance to shear rates up to 8.73 × 10^5^ s^−1^. Based on these results we used microbial CSTRs, operated at high revolution speeds and low aeration rates and found no negative impact on cell viability. Further, this cultivation approach led to substantially reduced gas flow rates, gas bubble and foam formation, while addition of pure oxygen was no longer necessary. Therefore, this study contributes to the development of more robust insect cell culture processes.

## Introduction

In the biopharmaceutical industry, products are mainly produced by cultivating organisms in suspension. A rather small share (only 2.3%) of all newly approved active pharmaceutical ingredients (APIs) are produced with insect cells as host. Insect cells produce recombinant proteins and virus-like particles (VLPs), a highly complex product class that is rapidly gaining importance^1^. In contrast, Chinese hamster ovary cells are the main work-horse in biopharmaceutical production; they produced 51% of all newly approved APIs from 2014 to 2018^2^. Based on fermentation technologies and operating strategies developed for CHOs, a broad portfolio of methodologies is available, which can be transferred to insect cell processes with only minor modifications. The system of choice for cultivating insect cells in suspension is the CSTR. These bioreactors can be either stainless steel, multi-use systems, with up to 25 m^3^ of working volume^3^, or a single-use bioreactor with up to 2 m^3^ of working volume^4^.

If the aim is to transfer methods and approaches developed with CHO to insect cell culture, the significant differences in oxygen demand between these cell types must not be neglected. *Spodoptera frugiperda* (*Sf9*) cells show four and *Trichupulsia Ni* (*Hi5*) even 13 times higher specific oxygen consumption rates than CHO cells, even though for CHO the literature values vary about one order of magnitude^5,6^ and these numbers even increase by 30–40% after the culture is infected^7,8^_._ The high oxygen demand of insect cells can cause severe problems in the cell culture process especially if the frequently postulated shear sensitivity for insect cells is really true.

The theory that mammalian or insect cells are highly sensitive to shear is widespread and persistent, although there is no sound scientific base supporting this hypothesis. On the contrary, there are studies, especially on CHO cell culture, which show that the influence of shear forces on the viability of CHO cells has been greatly overestimated. It has also been shown that cell damage will not occur as long as the size of a biological entity is less than the Kolmogorov scale of turbulence^9^. Different methods for quantifying the effect of hydrodynamic forces on mainly animal cells, are found in literature. They range from flow chambers containing a nozzle, rheological instruments, capillary tubes to specially designed flow devices where the applied shear is simulated via computational fluid dynamic (CFD)^10–15^ For insect cell lines little or no objective data are available. Goldblum et al. made attempts to determine the sensitivity under laminar shear conditions using a modified Weissenberg rheogoniometer^16^ and Ma et al. determined the sensitivity of Sf9-cells against hydrodynamic forces in a microfluidic channel^10^. Consequently, existing knowledge and concepts for insect cells are largely based on historical, empirical data, “rule of thumb”^17^, and experience with similar cell types.

The state-of-the-art approach in insect cell culture, is still based on the hypothesis of insect cells high shear sensitivity and consequently CSTRs used for cultivation are designed to maintain low shear forces caused by stirring. Their typical height to diameter (H/D) ratios are in the range of 1.5–2:1^18^; the standard gas transfer coefficient, k_L_a, ranges from 5 to 10 h^−1^; and the specific power input ranges from 5 to 300 W m^−3^^19^. These bioreactors are additionally operated at low stirring speeds that just meet the requirements for mixing. The oxygen transfer, on the other hand, is exclusively manipulated via the flow rate and the composition of the supplied gas. However, this type of process operation leads to a number of problems. High gas flow rates create more gas bubbles in the system, which in turn lead to increased foaming and shear due to the bursting of air bubbles on the liquid surface^20,21^. Low stirring speeds, in turn, can lead to mixing problems and thus increase the formation of zones and gradients—a problem that is particularly relevant in scale-up^22^. With limited mixing, the addition of pure oxygen to the aeration gas can cause oxidative stress in the cells, which in turn can affect process efficiency^23–27^.

In summary, the problems described in insect cell culture processes can all be linked to the assumption that these cells are very sensitive to shear stress. However, based on the published data on the shear sensitivity of mammalian cells, there is reason to question the extent to which there are any limitations in the field of insect cell culture process design related to shear sensitivity. Previous studies have shown that insect cells are not necessarily more shear sensitive than mammalian cells and since they are about the same size, the Kolmogorov approach would also point in this direction^28,29^. In this study, focused on the TN42 cell line, a cell line not yet well characterized yet, we decided to take a closer look on shear sensitivity. As a reference, we used CHO cells and SF9 cells, which are much better characterized. We applied a microfluidic shear device^30^ that could quantify the shear resistances of cells under controlled conditions without the need of CFD modeling. We applied different shear rates to insect and CHO cells to determine the tolerable range of shear for these cell types and we observed high shear resistance for all cells tested. Based on these results, the cultivation strategy for insect cells was completely redesigned. We applied high stirring speeds to ensure efficient oxygen transfer, in combination with low aeration rates to reduce shear forces triggered by bubble rupture and foam formation. We used microbial CSTRs, which differ dramatically from cell culture reactors, in terms of reactor geometry (H/D ratios: 2.5–3:1 or more), oxygen transfer capacity (k_L_a > 250 h^−1^, and may exceed 1000 h^−1^^31,32^), and specific power input (˃ 5 kW m^−3^^33–35^). The new process design based on the knowledge that insect cells can withstand quite high shear forces, eliminates many problems identified with previous insect cell culture processes and allows for high flexibility and better scalability.

## Materials and methods

### Cell lines

We purchased two High Five (ThermoFisher) insect cell lines: the BTI-TN-5B1-4 cell line and the Tnms42 (TN42) cell line (BTI, Gary W. Blissard), which  is alpha-nodavirus-free, TN-5B1-4 derivative^36^. In addition, we acquired a host cell variant of CHO‑K1 (ATCC CCL‑61) that was adapted to serum-free medium^37^ (Antibody Lab GmbH, Vienna, Austria). The stable CHO-K1/D1 clonal cell line produced an IgG1 antibody that specifically recognized tumor necrosis factor alpha.

### Cloning and generating recombinant baculoviruses and the virus stock

We used a baculovirus that encoded the hemagglutinin (HA) protein of Influenza virus A/California/04/2009 (H1N1) (GenBank accession no. JF915184.1) and the matrix protein for the Gag-polyprotein (Gag) of type 1 human immunodeficiency virus (GenBank accession no. K03455.1). These recombinant genes were codon-optimized for expression in *Trichoplusia ni* (IDTdna, Leuven, Belgium). After PCR amplification, the HA of H1N1 was inserted into the pACEBac-1 acceptor vector (EMBL, Grenoble), which resulted in pACEBac-1-H1. Similarly, the Gag fragment was cloned into the pIDC donor vector (EMBL, Grenoble), which resulted in pIDC-Gag. A Cre-LoxP recombination of the acceptor and donor vectors resulted in H1-Gag acceptor–donor fusion plasmids. The H1-Gag fusion plasmid was transformed into either *E. coli* DH10EMBacY (EMBL, Grenoble) or DH10EMBacp6.9Y cells, which harbored a yellow fluorescent protein (YFP) expression cassette under the control of the polH or p6.9 promoter, respectively. The purified bacmid DNA was transfected into *Sf*9 cells with the FuGene HD transfection reagent (Promega, Madison, Wisconsin, USA) according to the manufacturer’s instructions. Viral titers were raised by subsequent passaging, and the titer of the passage 3 stock was determined by measuring the half-maximal tissue culture infective dose (TCID_50_).

### Shear experiments

**Figure 1:**
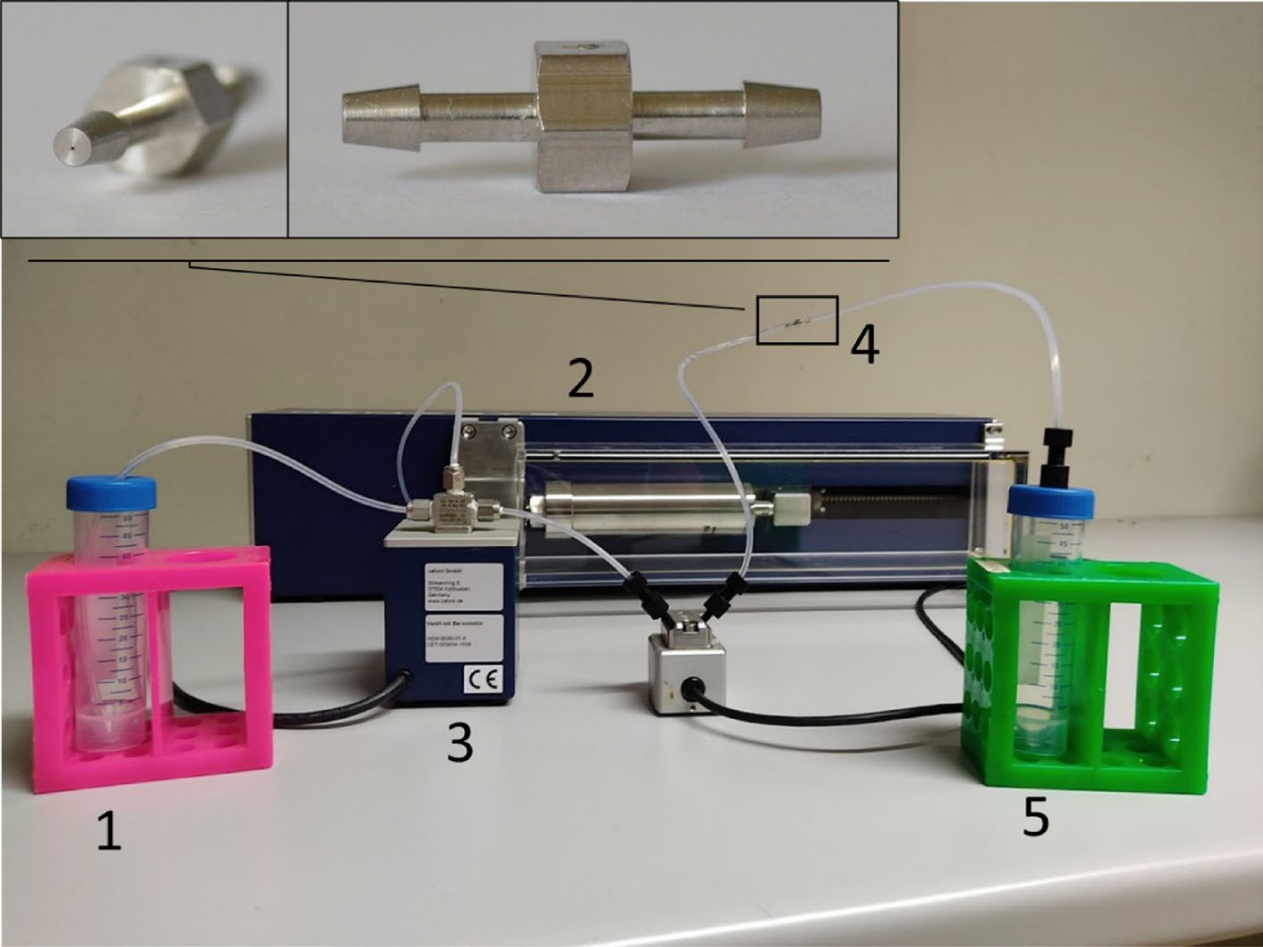
Illustration of the cell-stressing setup. (1) Sample reservoir, (2) syringe pump, (3) 2-way valve, (4) orifice, (5) sample collector. In the top left corner, the two insets display magnifications of the orifice (4).

The shear sensitivity and resistance of cells in suspension were tested with a shear device developed by Duerkop et al.^38^. This device was a T-4-SS micro-orifice (O´Keefe Control Co., Monroe, CT, USA) with a 15-fold reduction in diameter (from 1/16″ to 99 µm on a total length of 330 µm). It previously generated shear rates up to 10^8^ s^−1^ when used to evaluate the shear sensitivity of proteins^38^. We modified the described method by using a Nemesys XL syringe pump (Cetoni GmbH, Korbussen, Germany), instead of an ÄKTA P100 piston pump, to reduce pump induced cell stress. Figure 1 illustrates the experimental setup. With a two-way valve (3), the cells could either be hoovered from the sample reservoir (1) into the syringe pump (2) or, when the valve was switched, cells were pumped from the syringe pump (2) through an orifice (4), and into the sample collector (5).

Cells were pumped through the orifice at different volume flow rates. By increasing the volumetric flow rate, the shear rate was increased. We calculated the average and maximum shear rates (*γ*), as follows:

1 $$\gamma _{{{\text{average}}}} {\text{ = }}\frac{{16\nu }}{{3{\text{d}}}}$$

2 $$\gamma _{{{\text{max}}}} {\text{ = }}\frac{{8\nu }}{{\text{d}}}$$

where *v* is the velocity (m/s), and *d* is the diameter of the orifice (m). Equations (1) and (2) are valid in laminar flow conditions, which were applied in these experiments. To investigate the effect of the device, the collected cells were counted, and viability was determined. A control experiment without the device was used to estimate the impact of the system setup itself.

### Shake flask cultivations

#### Insect cell cultivation

For each experiment, cells from adherent cultures were transferred to suspension at a starting concentration of 0.5 × 10^6^ cells/mL. Cells were then expanded to the required cell concentrations. For all experiments, the cells were maintained in the exponential growth phase at 27 °C in shaker flasks, agitated at 100 rpm, and passaged until they reached a cell concentration of 4 × 10^6^ cells/mL. Then, cells were grown in serum-free medium which contains poloxamer 188 (Hyclone SFM4Insect, GE Healthcare). Viable cell counts (VCC) were determined with the trypan blue stain method in an automated cell counter (TC20 Biorad).

#### CHO cell cultivation

To acquire the seed culture, cells were thawed from a working cell bank and cultured in Dynamis AGT Medium (A26175-01, Thermo Fisher Scientific, USA), supplemented with 8 mM l-Glutamine (25030081, Sigma-Aldrich, USA), 1:1000 Anti-Clumping Agent (01-0057AE, Thermo Fisher Scientific) and 0.7 mg/L G418 (108321-42-2, Thermo Fisher Scientific). For pre-cultures, cells were sub-cultured in Dynamis AGT Medium with 8 mM l-Glutamine every 3–4 days at 37 °C, in a humidified incubator (Heracell v10S 160, Thermo Fisher Scientific) with 5% v/v CO_2_ and agitated at 200 rpm on an orbital shaker (88881102, Thermo Fisher Scientific). Cells were diluted to a total cell count (TCC) of 1.5 × 10^6^ cells/mL in Dynamis AGT Medium before the shear stress tests.

### Bioreactor cultivation and setup

Experiments were performed in 1 L (BioFlo320 1L, Eppendorf) and 1.5 L (DASGIP SR1500 DLS, Eppendorf) bioreactors. No additional baffles were deployed in the bioreactors used. The only obstacles to the free swirling motion of the fluid phase in the vessel are described below.

The BioFlo320 bioreactor system (d_i_ = 12 cm, h = 23.9 cm) was equipped with one pitched-blade impeller (3 blades; 45°, d = 6 cm, h = 4 cm) sitting on the end of the stirrer shaft. It was further equipped with a pH and a DO sensor (d = 1.2 cm, h = 20 cm and 22 cm), a ring sparger (d = 6 mm), a harvesting and a sample taking pipe (d = 6 mm) and a thermowell (d = 7.8 mm).

The DASGIP bioreactor system (d_i_ = 10 cm, h = 30 cm) was equipped with a six-blade Rushton impeller (d = 4.5 cm) sitting on the end of the stirrer shaft. It was further also equipped with a pH and a DO sensor (d = 1.2 cm, h = 32.5 cm), an L-sparger (d = 6 mm), a harvesting and a sample taking pipe (d = 4 mm) and a thermowell (d = 6 mm).

The specific power input of the DASGIP bioreactor was as follows:

3 $$\frac{P}{M}=\frac{Np\times \rho \times {N}^{3}\times {d}^{5}}{M},$$

where Np is the power number, ρ = 1050 kg/L, N is the corresponding stirrer speed in rounds per second, d is the impeller outer diameter (m) and M the mass of the culture broth. The Np values were experimentally determined using a torque meter^39^, in a setup of no gas flow. The specific power inputs were calculated using Eq. (3) and are summarized in Table 1.

**Table 1:** Stirrer speeds with corresponding tip speeds and calculated specific power input.

Stirrer speed (rpm)	Tip speed (m/s)	Specific power input (W/kg)
100	0.24	0.01
200	0.48	0.07
300	0.72	0.23
600	1.45	1.85
800	1.93	4.37
1000	2.41	8.54

The BioFlo320 system maintained the dissolved oxygen level at 30%, and pure oxygen was supplemented when needed. The temperature was maintained at 27 °C, and the pH was monitored. This setup was chosen, because it was described previously^7,40^. The 1.5 L DASGIP bioreactor maintained the pH at 6.4 ± 0.05 with 25% phosphoric acid and 7.5% sodium bicarbonate.

### Bioreactor infection strategy

Cells were grown in the bioreactor in batch mode, until they reached a cell density of about 2 × 10^6^ cells/mL. Next, they were infected with the generated baculovirus stock, at a multiplicity of infection (MOI) of 1 and diluted with fresh media to a final density of 1 × 10^6^ cells/mL.

### Analytical methods: tissue culture infectious dose assay

The titer of virus stocks was determined by measuring the TCID50^41^, based on the detection of YFP fluorescence. Briefly, *Sf*9 cells were infected with serial dilutions of virus stock or supernatant samples of the different cultivations in a 96-well culture plate (Corning Incorporated, USA). Plates were incubated at 27 °C without agitation. After 4 days, the wells were inspected with a fluorescence microscope (Leica DMIL-LED).

## Results and discussion

### Shear resistance of cell-lines: shear device experiments

To evaluate the influence of shear on insect and CHO cell lines, we set up controlled shear conditions with a micro-fluid shear device. In the first step, we conducted a control experiment to test the influence of the syringe pump and tubing on cell viability. We filled the syringe pump with the cell suspension and pumped it through the flow path without the nozzle. These experiments were performed at maximum pump speed (45 mL/min), which was the speed used to fill the syringe pump with the cell suspension. We evaluated cell viability before and after this treatment.

Next, directly before each experiment, we prepared 50-mL batches of cell suspensions at cell concentrations of 1.5 × 10^6^ cells/mL. We used an untreated cell suspension with a defined cell concentration and viability as the reference sample (control). Then, a 20-mL aliquot of suspended cells was drawn into the syringe pump each passage and pumped through the device. The first 10-mL fraction of cells was discarded to exclude potential impurities from a former run. The fraction from 10 to 15 mL was used to evaluate cell viability. Each volume flow rate was measured in triplicate, if not otherwise indicated, and the system was flushed with media between volume flow rate changes. We selected three different volume flow rates (3, 5, and 10 mL/min) for shear rate determinations (Table 2). For all the tested flow conditions, a laminar flow profile was present inside the shear device; thus, we used Eqs. (1) and (2) for shear determinations. We also calculated the dimensionless shear, which was the product of the average shear rate and the incubation time. For proteins, it was assumed that, when the dimensionless shear exceeded 10^4^, the proteins would irreversibly aggregate^42^. Although this theory was previously shown to be false for a large set of proteins^30^, the result could be true for cells.

**Table 2:** Flow rates inside the shear device, with corresponding Reynolds number, maximum and average shear rates, and the dimensionless shear.

Flow rate (mL/min)	Reynolds number	Max shear rate (s^−1^)	Average shear rate (s^−1^)	Dimensionless shear
3	613.47	5.24E + 05	3.49E + 05	17.93
5	1022.45	8.73E + 05	5.82E + 05	17.93
10	2044.89	1.75E + 06	1.16E + 06	17.93

Furthermore, we showed that dimensionless shear-associated aggregation did not apply to these cells. The product of incubation time and shear rate was constant inside the shear device, because at higher flow rates, the incubation time was reduced by the same amount as the shear rate was increased. Hence, the dimensionless shear was constant, as long as the flow conditions remained laminar. However, at higher flow rates, the viability decreased. This decrease indicated that cell damage occurred when the shear rate exceeded the threshold that maintained a constant dimensionless shear.

**Figure 2:**
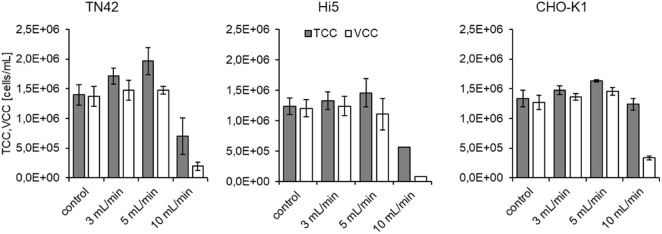
Total and viable cell counts after treatment with the shear device. Shear was measured at different flow rates for (left) TN42, (center) Hi5, and (right) CHO-K1 cells. Each run was performed in triplicate, except the Hi5 experiment (center) run at 10 mL/min.

Our experiments, conducted under controlled shear conditions, indicated that all three cell lines could withstand much higher shear rates than expected, based on the literature^43^. Figure 2 shows that all cell lines could withstand flow rates up to 5 mL/min, which imposed maximum and average shear rates of up to 8.73 × 10^5^ s^−1^ and 5.82 × 10^5^ s^−1^, respectively (Table 2). The increase in the TCC at flow rates of 3 and 5 mL/min could be attributed to the dispersion of cell clumps when passing through the orifice of the shear device. However, the shear imposed by 10-mL/min flow rates reduced the TCC and VCC. In addition, we observed a sharp increase in the VCC/TCC ratio. However, at lower shear rates, the shear had a positive effect, due to the dispersion of cell clumps^44^; clump dispersion also occurs when cells are filtered^45^.

### Bioreactor shear characterization

In a theoretical analysis, Sánchez Pérez, Rodríguez Porcel^46^ established the connection between the average shear rate ($\gamma$_av_) and the rotational speed of the impeller in turbulent flow. Below, Eq. (4) is based on a simplified assumption correlated to empirical data. In Eq. (5), the maximum shear stress (γ_max_) also took into account the media and type of impeller used.

The relationship between the stirring speed (N) in a CSTR and the average shear rate ($\gamma$_av_)^46^, for an A315 axial flow hydrofoil impeller, was:

4 $${\gamma }_{av}=33.1 \times {N}^{1.4}.$$

The relationship between the stirring speed (N) in a CSTR and the maximum shear stress ($\gamma$_max_)^47^ was:

5 $${\gamma }_{max}=3.3 x {N}^{1.5}x {d}_{i}{\left(\frac{\rho }{\mu }\right)}^{0.5},$$

where *µ* = 1.1 mPas, *ρ* = 1050 kg/m^3^ are related to the used media, and *di* = 0.06 m of the used pitched-blade impeller.

The calculated average and maximum shears, based on Eqs. (4) and (5) are shown in Table 3 for reasonable stirrer speeds that are typically used in bench-top bioreactors. According to these numbers, any stirring speed currently used in bioreactors would be below the critical value for the cell lines tested with the shear device. Consequently, shear generated in a bioreactor equipped with a stirrer using a pitched-blade impeller or a Rushton should never exceed the critical limit for insect cells.

**Table 3:** Stirring speeds and corresponding average (γ_av_) and maximum (γ_max_) shear rates for a 1 L bioreactor equipped with one pitched-blade impeller.

Speed (rpm)	γ_av_ (s^−1^)^a^	γ_max_ (s^−1^)	Dimensionless shear
100	6.77E + 01	4.16E + 02	2.34E + 07
200	1.79E + 02	1.18E + 03	6.17E + 07
500	6.44E + 02	4.65E + 03	2.23E + 08
800	1.24E + 03	9.42E + 03	4.30E + 08
1000	1.70E + 03	1.32E + 04	5.87E + 08
1500	3.00E + 03	2.42E + 04	1.04E + 09

^a^The average shear values were generated by Sánchez Pérez, Rodríguez Porcel^46^ with Eq. (5), for an A315 axial flow hydrofoil impeller (LIGHTNIN Mixers, Rochester, NY), which is similar to a pitched-blade stirrer.

Furthermore, when a cell cultivation lasts 96 h, the dimensionless shear would be up to 8 orders of magnitude above the shear observed in the shear device. that the concept of dimensionless shear was introduced around 1970^42^ and assumes, if proteins are incubated for a very long time, they will be harmed even by medium shear rates. If we assume that cells behaved like proteins, then cells incubated for long times should experience viability problems, even at rather low stirring speeds. We found that the maximum shear rate for short periods of time damaged cells, but an average shear rate for an extended period of time did not damage cells. Our findings indicate that cells can withstand high shear rates and that dimensionless shear should not be considered as critical. To reach the critical shear of 8.73 × 10^5^ s^−1^ the corresponding theoretical speeds for the used stirrer blades would be 16,380 rpm for the pitched blade and 19,850 rpm for the Rushton impeller. The microbial bench-top bioreactors used within this study have only a maximum stirrer speed of 1600 rpm. Hence, calculated maximum shear rates cannot be reached with this setup. Further, before cells would sense impeller-induced shear damage the effect of vortex generation, air entrapment, bubble collapse and cavitation at elevated impeller revolutions would be the main drivers for cell death and might incorrectly be attributed to impeller shear.

### Bioreactor shear experiments

The experiments with the shear device and the estimation of shear rates inside the bioreactor led to our conclusion that shear generated by stirring was not likely to damage insect cells. Because both insect cell lines showed similar behavior, we selected the TN42 cell line for the next series of experiments, which focused on verification of the shear device results.

**Figure 3:**
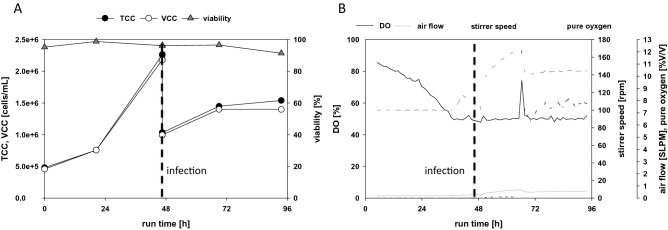
Reference process with TN42 insect cells. (**A**) Counts of total cells (TCC, filled symbols) and viable cells (VCC, open symbols), before and after infection (dashed line), and cell viability (triangles) over the course of the cultivation. (**B**) Trends are shown for the dissolved oxygen (DO)-level, the airflow, the stirrer speed, and the percentage of pure oxygen in the airflow. The time point of infection is indicated by the vertical bold dashed line.

In the first step, we conducted a TN42 reference cultivation run under standard operation conditions, in a 1 L BioFlo320 System (Eppendorf) equipped with one pitched-blade impeller. This experiment generated reference process data (Fig. 3).

In this reference experiment, cells were seeded at 0.5 × 10^6^ cells/mL and grown in batch mode until they reached 2 × 10^6^ cells/mL. At this point, the cells were infected with the baculovirus working virus stock at a MOI of 1, and they were diluted with fresh medium to 1.0 × 10^6^ cells/mL (Fig. 3A). The stirring speed ranged from 100 to 160 rpm corresponding to a maximum shear rate of 416 s^−1^ and 842 s^−1^, and the aeration rate ranged from 0.2 to 0.5 standard liter per minute (SLPM). The results showed that, even at a cell concentration of 1.5 × 10^6^ cells/mL, after infection, it was necessary to add pure oxygen to maintain the dissolved oxygen level at 30% and avoid high stirring rates. Infection caused a decline in the cell growth rate, and at 48 h post infection, cell viability was reduced to 91.4% (Fig. 3A).

**Figure 4:**
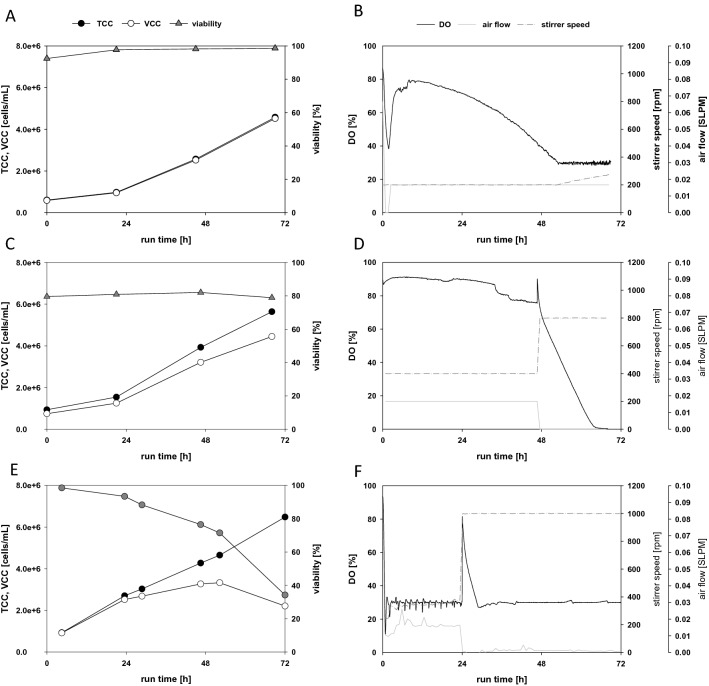
Batch fermentation of TN42 insect cells in a microbial bioreactor, at high stirring speeds. Bioreactors were equipped with either one Rushton impeller (**A**,**D**) or 3 levels of impellers (**E**,**F**). (Left column) Viability (triangles), and counts of total cells (TCC, filled circles) and viable cells (VCC, open circles) in the batch cultivation; (right column) the corresponding dissolved oxygen (DO, black solid line), stirring speed (dotted line), and air flow (grey solid line). Stirring speeds were (**A**,**B**) 200 rpm, incrementally increased to 270 rpm; (**C**,**D**) 400 rpm, stepped to 800 rpm at 48 h; (**E**,**F**) 400 rpm, stepped to 1000 rpm at 24 h.

According to Table 3, the shear in the reference setting was more than two orders of magnitude below the critical values determined in the microfluidic shear device experiments. Therefore, to introduce higher shear rates with stirring, we switched to a microbial bioreactor (SR1500DLS, Eppendorf DASGIP System) equipped with one Rushton impeller. Cells were grown in a 500 mL batch volume and the stirring speed was set to 200 rpm (883 s^−1^), as a starting value which corresponds to a calculated specific power input of 0.07 W/kg. The air flow was maintained at a constant 0.016 SLPM. Cells were seeded at 0.5 × 10^6^ cells/mL and grown in batch mode for 72 h without the addition of fresh media. The cell viability increased during the batch run, and the cell density reached 4.5 × 10^6^ cells/mL (Fig. 4A). Cell viability was not impacted by the shear rates generated with a Rushton impeller, even running at speeds up to 270 rpm (1385 s^−1^, 0.17 W/kg).

The next batch cultivation (Fig. 4C) was started at 400 rpm (2497 s^−1^, 0.55 W/kg), and a step increase to 800 rpm (7064 s^−1^, 4.37 W/kg) was applied after 48 h of cultivation. The aeration rate was set to 0.016 SPLM to minimize bubbles and foam formation, because no antifoam was used in this experiment. Although the inoculated cells showed low viability compared to the other batches (Fig. 4A), their viability increased in the first 48 h, from 79.6 to 82%. This increase in viability was also observed in the previous experiment (Fig. 4A), which led to the conclusion that a bioreactor powered by a Rushton turbine at 400 rpm would not impact the viability of insect cells. At the initial 400 rpm stirring rate and the low aeration rate, the DO level slowly decreased from 100 to 65% over the initial 48 h, and the culture reached a cell density of 4.0 × 10^6^ cells/mL during this time. At 48 h, the stirring speed was increased to 800 rpm, and unintentionally, the aeration rate was set to 0 SPLM for the last 24 h of the batch run. Consequently, the DO steadily decreased to 0% for the last couple of hours of the experiment, and cells started to die, due to limited oxygen.

Another cultivation was performed at a working volume of 1 L, but the reactor was equipped with three Rushton blades which was the setup we use for microbial cultivation. The initial stirring speed of 400 rpm was stepped to 1000 rpm (9872 s^−1^, 8.54 W/kg) after 24 h (Fig. 4E,F). At 24 h after this change, the viability initially decreased by about 5%; but at 48 h after the step to 1000 rpm, the viability dropped by 71.5%. This observation can be explained by the high stirring speed, which led to the formation of a liquid vortex, because no baffles were installed in the bioreactor. As a result, additional air was introduced into the suspension via the vortex surface, and the air was split into small bubbles by the Rushton elements. Thus, the air associated cell damage increased, due to bubbles bursting. The cells could not withstand these harsh conditions. Similar observations were previously described by Murhammer David and Goochee Charles^48^ and by Maranga et al.^49^.

#### New control strategy for insect cell cultivation processes

**Figure 5:**
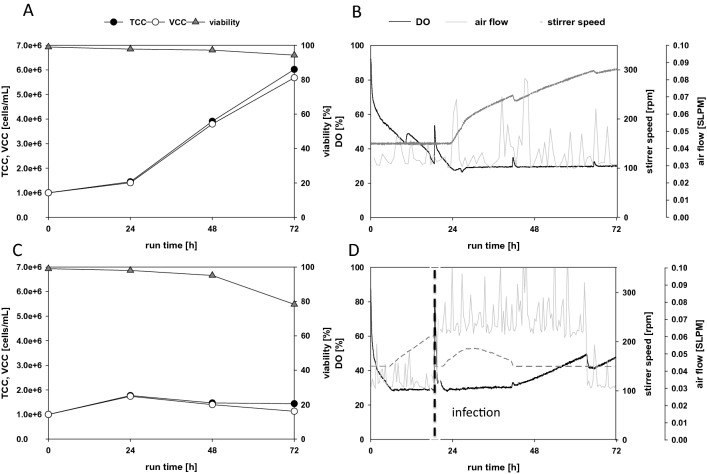
Testing a new control strategy on two cultivations of TN42 insect cells. The controller was tested on (**A**,**B**) an uninfected batch cultivation, and (**C**,**D**) an infected batch process. In both cases, the starting stirring speeds were 150 rpm, and the controller increased or decreased the speed to maintain the DO at 30%. (**A**,**C**) Counts of total (TCC, filled circles) and viable (VCC, open circles) cells, and cell viability (triangles) over the course of the batch cultivation. (**B**,**D**) Dissolved oxygen (DO, solid black line), air flow (solid grey line), and agitation speed (dotted line). The vertical line in Figure (**D**) indicates the time of infection.

With the information generated in the preceding experiments, we set up a modified DO control strategy in the microbial bioreactor. The goal was to maintain the gas flow rate as low as possible to minimize foam formation and bubble-associated shear/stress. The PID control strategy for maintaining the DO was adapted by linking the stirring speed, which was the main parameter, to the airflow rate. In the initial phase, the impeller speed was set to the minimum (150 rpm, 574 s^−1^, 0.03 W/kg). Then, when the DO level reached the set point of 30%, the controller was set to increase the stirring speed, incrementally, up to a maximum of 800 rpm. The stirrer was equipped with a Rushton impeller, and an L-sparger was used to distribute the aeration gas. During the cultivation of uninfected cells, the stirring speed increased to 300 rpm (1622 s^−1^, 0.23 W/kg), and at the end of the exponential growth phase, the TCC was 6 × 10^6^ cells/mL (Fig. 5A). The gas flow rate was set to the minimum of 0.03 SLPM, but unfortunately, the controller could not maintain this precise rate (Fig. 5B,D).

In parallel, the same process control strategy was tested in a batch that received a virus infection at 24 h. Due to the infection and VLP production, cell growth stopped (Fig. 5C), but oxygen consumption continued to increase (Fig. 5D). Additionally, at the timepoint of infection, the aeration rate increased to 0.06 SLPM. Compared to the reference process for the infected batch (Fig. 3), in this setup, there was no need to add pure oxygen, because increasing the stirring speed provided efficient oxygen transfer. The results of these batch cultivations are in line with Kioukia et al., where infected Sf9 cells were cultivated at 400 rpm with a similar stirrer without an influence in production^28^.

#### CHO cultivations

**Figure 6:**
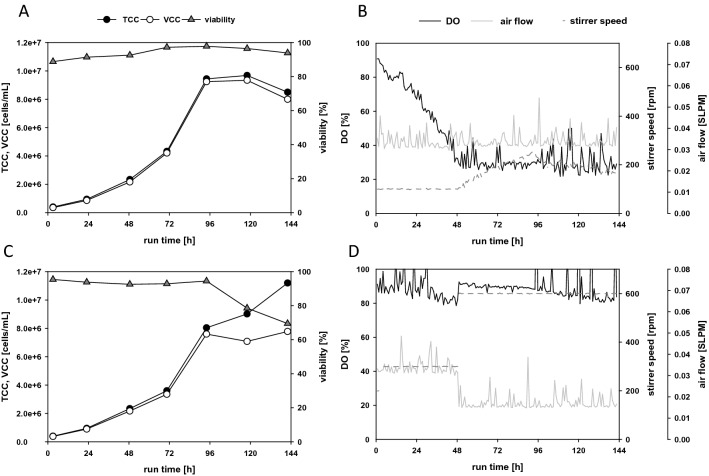
The impact of a high stirring speed tested by comparing two cultivations of CHO cells in microbial bioreactors, at different stirring speeds. (**A**,**B**) A standard batch cultivation with a low stirring speed (100 rpm); (**C**,**D**) a batch process with a high stirring speed (300 stepped to 600 rpm). (Left column) Viability (triangles), and counts of total cells (TCC, filled circles) and viable cells (VCC, open circles) in the batch cultivation; (right column) the corresponding dissolved oxygen (DO, black solid line), stirring speed (dotted line), and air flow (solid grey line).

To determine the impact of high shear due to increased stirring speeds, we performed a direct comparison between two CHO batches cultivated at different stirring speeds in a 1.5 L microbial CSTR bioreactor (SR1500DLS, Eppendorf) with a working volume of 500 mL. One bioreactor (Fig. 6A,B) was operated at a low stirring speed, starting at 100 rpm, and the aeration rate was set to 0.03 SLPM. The DO was maintained at 30% by incrementally increasing the stirring speed, which mimicked a standard CHO batch cultivation. The second bioreactor was operated at an increased stirring speed. After a short adjustment phase at 200 rpm, the stirring was maintained at 300 rpm with an aeration rate of 0.03 SLPM. Then, after 48 h, the stirring was increased to 600 rpm, and the aeration rate was lowered to 0.016 SLPM (Fig. 6D). In both reactors, the sparger supplemented the medium with carbon dioxide to control the pH.

We found that cell growth rates behaved nearly the same at the low and high stirring settings (Fig. 6A,C). Moreover, viability was not influenced by the high stirrer speed during exponential growth, which ended at around 96 h after inoculation. At the high stirring speed, the DO never dropped below 80% throughout the entire process (Fig. 6D). However, we observed a difference in viability during the stationary phase; the viability decreased more rapidly in the bioreactor with the faster stirring speed (Fig. 6C,D) than in the bioreactor with the slower speed (Fig. 6A,B). This could possibly be due to the high DO levels during the batch cultivation. It has been reported that high DO levels during a cultivation can lead to oxidative stress and also to reduced protein yields^23–26^.

Overall, our results showed that CHO cells could be cultivated with a Rushton impeller which was also shown by Ref.^27^ but at higher stirring speeds, without damaging the cells. Additionally, aeration rates could be maintained at a minimum, and it was not necessary to add pure oxygen throughout the process. With the higher stirring rate and the lower aeration rate, foaming was nearly eliminated during the batch cultivations; thus, no antifoam had to be added. Applying this modified DO control strategy could enable CHO cell cultivations to achieve higher cell concentrations.

## Conclusion

The shear device described in this study was an efficient and simple tool to apply defined levels of shear directly to cells to characterize their shear sensitivity. In this study, we used the shear device with insect and CHO cells, but it can also be used for other cell lines, viruses, or VLPs to determine critical shear stress.

We found that with the shear device methodology both insect cell lines High Five and the Tnms42 as well as the CHO-K1 cell line could withstand maximum and average shears of 8.73 × 10^5^ s^−1^ and 5.82 × 10^5^ s^−1^, respectively. These results are in full agreement with the finding of a much higher robustness of different cell types to shear forces published by Nienow et al.^29^. From our point of view, it is time to dispel the myth about the high shear sensitivity of cells, as this leads to massive and unnecessary limitations in process control.

Knowledge of the critical shear for the cell types investigated facilitated the design of a new DO process control regime, based on high energy input through stirring, with applying typical Rushton-powered microbial bioreactor setup. With this setup, the oxygen transfer rate could be significantly increased, even at very low gas flow rates. As expected from the micro-fluidic experiments, high stirring speeds did not harm neither the insect cells nor the CHO cells, as long as the gas flow rate and bubble introduction were maintained at low levels. In addition, low aeration rates provided significantly reduced foam formation, which was beneficial for both the process and the cells. The cell densities achieved in this study required maximum stirring speeds of 300 rpm (1622 s^−1^, 0.23 W/kg) for insect cells and 220 rpm (1019 s^−1^, 0.09 W/kg) for CHO cells. These stirrer speeds introduced shear rates that were far below the previously described critical values. The reduced cell viability observed in the experiment with stirring at 1000 rpm was most likely caused by vortex formation, which can, to a certain extend be prevented by introducing baffles. Our results showed that the oxygen transfer rates that we achieved with relatively high stirring with Rushton-powered reactor design at low aeration rates produced much higher cell densities than the conventional operational mode. Further, our setup significantly reduced gas flow rates and avoided the application of pure oxygen.

Our control regime has the advantage of improving the economic efficiency of the process, but more importantly, the lower gas volumes in the suspension also reduces foam formation and bubble rupture at the liquid surface. This phenomenon can positively affect cell viability, virus quality, and products, like for instance, VLPs.
